# Certain Points Connected with the Structure of Dentine

**Published:** 1888-12

**Authors:** F. J. Bennett


ARTICLE II.

### CERTAIN POINTS CONNECTED WITH THE STRUCTURE OF DENTINE.

BY F. J. BENNETT, M. R. C. S., L. D. S., ENG.

In one of the numerous and interesting contributions to molecular physics in March, 1885, Dr. W. M. Ord stated that it was possible to etch the surface of pieces of ivory and mother-of-pearl by immersion in a solution of subcarbonate of potash in glycerine. A year previously he (Mr. Bennett) had attempted to extend some of the well-known experiments of Rainey on the formation and disintegration of calculi with a view to obtaining artificial absorption and deposition in teeth and bones. Dr. Ord's paper came, therefore, as a welcome addition to his knowledge on the subject. He made various experiments in order to apply this method to the dental tissues, first using subcarbonate of potash in glycerine, and bicarbonate of soda, also in glycerine. At the same time, in order further to check this action, specimens were placed in pure glycerine alone. To his surprise, in both cases such similarity resulted that he was led to the conclusion that the real active agent in the decomposition must be glycerine alone, and since then he had used it exclusively.

His method of procedure was: (1) To grind and polish freshly extracted teeth sufficiently thin for microscopical purposes, then suspend them in pure glycerine, or over one of the compounds mentioned, for periods of from one to six months or longer, then wash and mount in glycerine for examination. (2) Freshly extracted teeth were immersed whole in pure glycerine for similar periods, then ground, polished and mounted as before. (3) Whole teeth were placed in very dilute solutions of glycerine, the strength of which was daily increased until pure glycerine was used, the specimens being kept in this for one or two months.

Examination with the naked eye showed that with pure glycerine alone there was neither obvious loss of tissue nor rigidity, the chief feature being the increased translucency of the dentine. On examining one of the sections with a low power under the microscope, taken from a young tooth, longitudinal to and including the pulp cavity, but without the pulp tissue, no conspicuous change was observable excepting in the dentine bordering on the pulp cavity, which presented a fringed and slightly laminated appearance. Under a higher power this appearance proved to be due to the loss of the intertubular tissue, leaving the dentinal tubes clearly defined; but this removal had not entirely freed the tubes. Their course appeared interrupted at regular intervals by layers of membrane having a parallel direction to the surface of the pulp, the layers bearing a general resemblance to that seen in interglobular dentine. Circular apertures, however, not solid globules, occupied the surface of the membrane; oval spaces were also found between the layers of the membrane. Through these apertures dentinal tubes could be seen crossing from one layer of membrane to another, and completely freed of their intertubular substance; the tubes seemed to arise from the upper and under surface of the membrane, and in places arose distinctly as processes from the margins of the membrane, in such a manner as strongly to resemble irregular cells and processes. A characteristic feature, moreover, was the regularity with which the tubes were measured off in short lengths by the crossing of the membrane. The specimens varied, not so much as to the time they had been immersed before mounting, but as to the time they had been mounted; for if a drop of glycerine were added beneath the cover glass from time to time, the specimen continued to be acted upon indefinitely. In this way fresh layers of membrane and additional lengths of tube could be brought into view. He was of opinion, however, that in other respects specimens were best examined early, many seeming contradictory and anomalous appearances, in his judgment, being due to specimens being mounted too long or allowed to come in contact with air.

Having become familiar with the various appearances assumed by the membranous layers as seen in profile, he selected a specimen presenting to view a continuous and fairly flat surface of the young layer of dentine parallel to the pulp, taken from the inferior fang of a lower molar. In the centre, the dentinal tubes were seen directly cut across; as the sides were approached they became gradually more oblique. Besides the appearance of layer succeeding layer, before described, over large areas scattered cells appeared to have taken the place of the membrane. These cells, seen in every variety of position, were generally of an elongated stellate shape. They were best seen where the dentinal tubes were oblique in direction; from this point to where the tubes were cut direcdy across, a curious and at first puzzling, appearance was presented. The uniform distribution of the dentinal tubes seemed broken by little gaps, as though they had been pushed aside. After careful examination, he concluded that these apparent gaps were cells similar in nature to those seen at the margins and other parts of the specimens, where they presented the more familiar aspect of stellate cells.

In connection with the appearances of membranes in the region of the pulp cavities, he might remind them that long ago it was pointed out by Salter and others that the interior of the cap of forming dentine on examination revealed an appearance of structure similar in its relations to interglobular layers of dentine. This he regarded as strong confirmatory evidence of the effect of glycerine being non-destructive in its action, since it revealed appearances so exactly resembling the natural ones. From the appearances which the specimens presented, it was manifest that the glycerine had acted in a selective manner, removing certain portions of the tissue only.

Various explanations might be given of the appearances described. First, it might be assumed that the membranes merely represented a part of the matrix itself, which possessed a greater power of resisting the action of the glycerine than the rest. It might further be assumed that the surface of the membrane itself presented unequal stages of calcification, so that portions of it had become removed, leaving behind the circular apertures. If this were true it might fairly accord with the theory of globular calcification in dentine. This explanation might be even strengthened by the fact that the effect of submitting interglobular dentine to the action of glycerine is to bring out exclusively and clearly the membranes which surround the globules. Further, it might be considered that the glycerine had produced separation of the layers of the matrix through the unequal expansion or contraction of certain parts. Upon this point, however, Dr. Lionel Beale had spoken very positively; he said that all tissues, both hard and soft, neither shrink nor swell in the strongest glycerine, if only the glycerine were first diluted and then gradually increased in strength. In order, however, to test the expansion of a substance almost identical in nature with the animal basis of dentine, he (Mr. Bennett) immersed a strip of gelatine in glycerine for some weeks, and a strip of gelatine was also immersed in water. At the end of the period, that in glycerine had not expanded or altered in measurement, while that in water had become softened and swollen in all directions. A third assumption would be to regard the appearances as evidence of cell structure.

Proceeding to describe the dentine in calcified pulps of old teeth, where much variety occurred, the author of the paper said, not only did the effects of glycerine show themselves well in those situations, but from the fact that it was impossible to preserve, in ground and polished specimens, both the dentine and parts of the semi-calcified pulp, he was yet able to obtain specimens showing cell structure and mounted directly in Canada balsam, which served well as a standard of comparison by which to judge of the distorting effects, if any, of glycerine. The result of these comparisons was greatly in favor of the glycerine, which brought out the structures with greater clearness and removed the calcified portions, bringing into view the parts which underlie them. The appearances of specimens in which the dental pulp was also present he would leave to be described on another occasion, likewise the consideration of the appearances of enamel under glycerine, in order that he might add a word as to the effects of this reagent on the cementum.

This tissue, as was natural from its containing the smallest proportion of lime salts of any of the hard structures of the teeth, was the most completely affected by glycerine, so thoroughly, indeed, that almost every trace of it could be removed by absorption. The absorption, however, was usually most visible in specimens which had been ground and mounted for sometime.

Briefly to summarise the results of his investigation: first, it had established the fact that glycerine was capable under certain conditions of decomposing the hard tissues of the teeth, and that it probably did so by the removal of the intercellular substance or matrix, where it existed in a certain state of density; thus, in the cementum, the least dense of the three tissues, it was most active, the cells being loosely united were unable to hold together, and disappeared with the surrounding decalcification; in the dentine, a tissue which there was reason to believe increased in density in the order of its deposition, the younger layers were chiefly affected. He need hardly point out, in conclusion, that beyond furnishing a new and valuable re-agent wherewith to study the tissues and furnish fresh data from which to reconsider certain points in the structure of dentine and cementum, there was that other aspect in which glycerine, from the manner of its absorption of the tissues artificially, might lead a step onwards to a better understanding of complex and hidden methods employed by nature herself.

Dr. ORD: I rise, sir, in obedience to your request; and first let me congratulate Mr. Bennett upon the ability and suggestiveness of his paper. In the beginning he alluded to some papers of my own as bearing, from his point of view, upon his researches—which I may say I think do him great credit;—they are scattered papers and cover a good deal of ground. I hardly know Sir, how far I may trespass upon the time of the Society, but I will not be very prolix. It happened two or three years ago that Surgeon-Major Bidie, now Sanitary Commissioner of Madras, wrote a letter to *Nature* to say that he had observed that certain glass vessels upon which white ants' mud had been deposited became etched over that portion which had been so covered, and he asked if any acid was to be found in ants' secretions capable of acting on glass. It was known to me, from very long association with Mr. George Rainey that he had observed that glass surfaces were eroded when exposed to the action of carbonate of lime deposited in the presense of colloids. He had shown that a perfectly clean and perfectly smooth glass slide, after being placed for a time in a solution of gum with carbonate of lime, became perfectly dim, and was eaten out into little shallow round depressions. When carbonate of lime was deposited in gum it was seen taking, instead of the form of a rhombohedron, which it should take, a spherical form, and he demonstrated that the colloid annulling the polarities of crystallization allowed simple attraction to work unhindered, so that, where there should have been a crystal with plane surfaces and sharp edges, we got a sphere: where a colloid was formed on the surface of the glass it drew away molecule by molecule from the glass into itself forming a little bed in the shape of a slight hemispherical depression. I repeated Mr. Rainey's experiments, using gum, albumen, and glycerine together, and I obtained, after some length of time, specimens much more remarkable than Mr. Rainey's (simply owing to larger exposure) in the presence of a colloid and a perfectly neutral salt. It occured to me to try ivory and mother o'-pearl in a parallel series. I used, with regard to glass, glycerine and nascent carbonate of lime; but when I was dealing with carbonate of soda and glycerine in connection with ivory and mother-o'-pearl, although I used in some experiments no carbonate of lime, I still got marked etching. That, of course, raised a question of a different aspect, and I may say that these were not acted upon by carbonate of potash or carbonate of soda without glycerine. What I believe, then, to be the explanation is that whereas in the glass the etching had come about by "molecular coalescence," I thought we had here the evidence of "molecular disintegration." Let me explain these terms. Mr. Rainey, when he found spheres of carbonate of lime in gum, found that any two or more spheres coming into contact with one another coalesced gradually into one perfect sphere; this he called "molecular coalescence." When such spheres—really little pearls—were placed in a fresh solution of gum, they underwent dissolution, the earthy matter being drawn out of them, leaving only a ghostly sphere of the organic matrix. This Mr. Rainey called "molecular disintegration." The contact of the glycerine and carbonate of potash with the surface of the ivory or mother-o'-pearl introduced a new colloid, and it would in like way disturb the original arrangement, draw out the earthy matter, and leave the matrix behind. This is the way in which I have explained the appearances. Mr. Bennett, in his experiments, has eliminated the carbonates and dealt only with glycerine. It is very interesting to notice that in proportion as the structure was less compact he has more readily drawn out the earthy material from its organic matrix, and where the organic matrix is more completely formed and more completely solid, he has left the organic structure well preserved, almost as if he had dissolved the earthy matter with an acid.

As to the cells, I will not express an opinion, except that I think these are re-arrangements of the earthy matter. However, he seems to have hit upon something which may be of great value in such researches as he is pursuing. He seems able from solid teeth to withdraw the inorganic without disturbing the organic matter. This, at least, seems to be the explanation of his interesting results. I should not like to say more until the specimens have been examined by polarized light to see whether they be organic spheroids or inorganic matter. While thanking you for your kind attention, I must express my gratitude to Mr. Bennett for putting us on a new line of observation.

Mr. F. J. BENNETT: May I be allowed, sir, to break through the rules, and reply to Dr. Ord at once? as I believe he has to attend another meeting. There is no doubt in my own mind that the appearances of structure exhibited are not due to re-arrangement of the matrix. There is undoubted proof that it really represents a natural structure which we had not hitherto known to exist. We have similar examples in interglobular spaces, and there are other instances. Salter says if the inside cap of young forming dentine be examined, it will be found to possess a cellular appearance, very much that of interglobular dentine, and the appearances in my specimens are very similar. With reference to polarized light, I have submitted my specimens to it and there is no doubt, after analysing them, that they are calcified structures. I think that those who wish to thoroughly follow up Dr. Ord's investigations will do well to read his own works; they are very interesting, and form a sort of second chapter to the leading works on this subject.—*Transactions of Odontological Society.—London Dental Record.*

